# Exploitation of the interaction of measles virus fusogenic envelope proteins with the surface receptor CD46 on human cells for microcell-mediated chromosome transfer

**DOI:** 10.1186/1472-6750-10-37

**Published:** 2010-05-06

**Authors:** Motonobu Katoh, Yasuhiro Kazuki, Kanako Kazuki, Naoyo Kajitani, Masato Takiguchi, Yuji Nakayama, Takafumi Nakamura, Mitsuo Oshimura

**Affiliations:** 1 Division of Human Genome Science, Department of Molecular and Cellular Biology, Faculty of Medicine, Tottori University, Yonago 683-8503, Japan; 2 Chromosome Engineering Research Center, Tottori University, Yonago 683-8503, Japan; 3 Department of Biomedical Science, Institute of Regenerative Medicine and Biofunction, Graduate School of Medical Science, Tottori University, Yonago 683-8503, Japan; 4 Division of Functional Genomics, Research Center for Bioscience and Technology, Tottori University, Yonago 683-8503, Japan; 5 Core Research for Evolutional Science and Technology (CREST) project, Japan Science and Technology Agency, Kawaguchi 332-0012, Japan; 6 Core Facility for Therapeutic Vectors, The Institute of Medical Science, The University of Tokyo, Tokyo 108-8639, Japan; 7 RNA and Biofunctions, Precursory Research for Embryonic Science and Technology (PRESTO) project, Japan Science and Technology Agency, Kawaguchi 332-0012, Japan

## Abstract

**Background:**

Microcell-mediated chromosome transfer (MMCT) is a technique by which a chromosome(s) is moved from donor to recipient cells by microcell fusion. Polyethylene glycol (PEG) has conventionally been used as a fusogen, and has been very successful in various genetic studies. However, PEG is not applicable for all types of recipient cells, because of its cell type-dependent toxicity. The cytotoxicity of PEG limits the yield of microcell hybrids to low level (10^-6 ^to 10^-5 ^per recipient cells). To harness the full potential of MMCT, a less toxic and more efficient fusion protocol that can be easily manipulated needs to be developed.

**Results:**

Microcell donor CHO cells carrying a human artificial chromosome (HAC) were transfected with genes encoding hemagglutinin (H) and fusion (F) proteins of an attenuated Measles Virus (MV) Edmonston strain. Mixed culture of the CHO transfectants and MV infection-competent human fibrosarcoma cells (HT1080) formed multinucleated syncytia, suggesting the functional expression of the MV-H/F in the CHO cells. Microcells were prepared and applied to HT1080 cells, human immortalized mesenchymal stem cells (hiMSC), and primary fibroblasts. Drug-resistant cells appeared after selection in culture with Blasticidin targeted against the tagged selection marker gene on the HAC. The fusion efficiency was determined by counting the total number of stable clones obtained in each experiment. Retention of the HAC in the microcell hybrids was confirmed by FISH analyses. The three recipient cell lines displayed distinct fusion efficiencies that depended on the cell-surface expression level of CD46, which acts as a receptor for MV. In HT1080 and hiMSC, the maximum efficiency observed was 50 and 100 times greater than that using conventional PEG fusion, respectively. However, the low efficiency of PEG-induced fusion with HFL1 was not improved by the MV fusogen.

**Conclusions:**

Ectopic expression of MV envelope proteins provides an efficient recipient cell-oriented MMCT protocol, facilitating extensive applications for studies of gene function and genetic corrections.

## Background

Microcell fusion is a method which enables the transfer of a single mammalian chromosome or its fragment from donor to recipient cells. This method consists of five essential steps: 1) micronucleation of donor cells; 2) enucleation of the micronucleated cells; 3) isolation of microcells; 4) fusion of microcells with recipient cells; and 5) selection of viable microcell hybrid clones. Microcell-mediated chromosome transfer (MMCT) offers several advantages for the transfer of genetic material between mammalian cells. Thus, megabase-sized stretches of an intact chromosome can be moved, which tend to be stable and freely segregating in recipient cells [1]. MMCT to patient-derived cells, followed by functional complementation assays, has been used for the genetic mapping and identification of genes responsible for hereditary recessive disorders and for tumor suppressor genes [2-5]. Other fields have taken advantage of MMCT for addressing genomic instability, genomic imprinting, chromatin modification, and structural and spatial organization of the genome [1,6-9]. Transfer of human chromosome fragments or artificially engineered chromosomes into embryonic stem cells has also successfully produced transchromosomic (Tc) animals [10,11]. These Tc animals have been used as sources of therapeutics and as models of human disorders such as Down's syndrome [2,12,13]. MMCT has also been applied to construction and manipulation of artificial chromosome vectors for potential human gene therapies [14,15]. MMCT has thus paved the way for the use of mammalian chromosomes as gene delivery vectors.

The most commonly used reagent for microcell fusion is high molecular weight polyethylene glycol (PEG) [16]. Since the establishment of standard method in the 1980s, the MMCT has been achieved with a specific class of recipient cells, for which the introduction of considerable changes in the fusion protocol was not necessary. However, little is known about the fusion mechanism of PEG. PEG may cause the redistribution of intramembrane molecules within the plasma membrane. This ability of PEG has been attributed to the ordering of water induced by a high concentration of polymer [17]. As well as inducing cell fusion, the PEG treatment concomitantly results in extensive cell damage and loss of cell viability because of the induced cytotoxicity [18]. Sensitivity to the PEG cytotoxicity is known to be cell type-dependent and is regulated by the lipid composition of the cell membrane [19,20]. Consistent with these data, the success of microcell fusion by PEG seems to depend on the particular combination of donor and recipient cells. Characterization and engineering of the lipid composition of cell membranes in order to avoid the PEG cytotoxicity is an intriguing but daunting task [21]. Therefore, to exploit novel applications of the MMCT, a more efficient, less toxic and more easily manipulated fusion protocol than PEG treatment needs to be developed.

**Figure 1:**
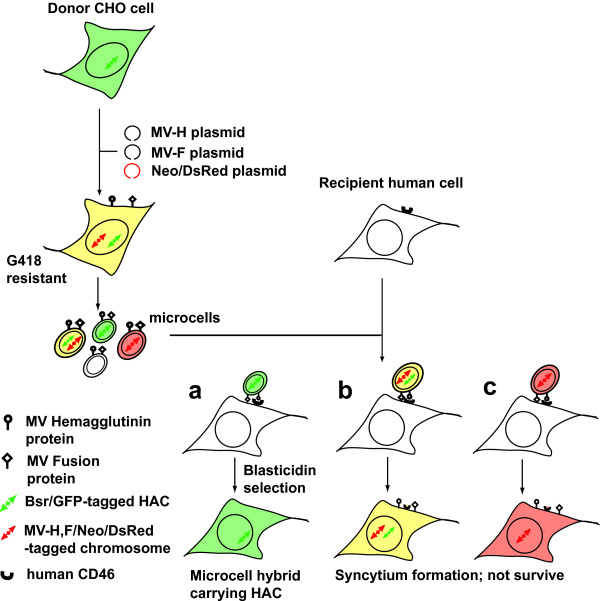
**Rationale for microcell fusion using an MV fusogen**. Donor CHO cells carry a human artificial chromosome (HAC) tagged with blastcidin-resistant (Bsr) and GFP gene. The CHO cells are transfected with plasmids encoding the MV-Fusion (MV-F) and Hemagglutinin (H) proteins and selection marker (Neo/DsRed). Microcells are prepared from the G418-resistant CHO donors and gave to recipient human cells. They are commonly coated with MV fusogen but contain different chromosomes. The donor-derived chromosome within the microcell is donated to the recipient cells by microcell fusion, which is mediated by interaction of the MV fusogen and the CD46 receptor presented on the surface of recipient cells. (a) The bsr-tagged HAC is rescued in mycrocell-hybrid by selection culture with Blasticidin. (b, c) On the other hand, introduction of the MV-H/F-tagged chromosome into recipient cells results in de novo synthesis of H/F proteins, leading to cell death caused by syncytium formation with the surrounding cells.

One well-documented naturally occurring membrane fusion event is that which occurs during the infection of host cells by enveloped viruses. The measles virus (MV), which causes the acute contagious disease of measles, has an envelope protein complex that is used for both virus attachment and membrane fusion [22]. The complex is composed of two integral membrane proteins, the hemagglutinin (H) and fusion (F) proteins. MV-H is the transmembrane glycoprotein responsible for the interaction of the virus with its cellular receptors. Two receptors have been identified for the live attenuated Edmonston B vaccine strain of MV (MV-Edm): the ubiquitously expressed regulator of complement activation CD46 and the lymphocyte-specific protein of the immunoglobulin (Ig) superfamily, SLAM. The H protein mediates attachment to either one of these cell surface receptors, and signals to the F protein to trigger membrane fusion. Infected MV propagates both by virus release and reinfection, and by cell-cell fusion. Virus-induced cell-cell fusion plays an important role in the propagation and pathogenicity of MV. MV-Edm has been shown to be selectively oncolytic [23]. This effect is due to the fact that CD46 is upregulated in tumor cells, thereby allowing MV-Edm to selectively target and destroy tumor cells through this receptor [24]. Furthermore, MV-Edm has been retargeted by engineering the H protein to efficiently enter tumor cells via alternative cellular receptors [25,26]. These previous studies prompted us to test the idea that transduction of genes encoding the H and F proteins into non-human donor cells may lead to the production of fusogenic microcells (Figure 1).

In this study, we tested the feasibility of using the MV viral fusion machinery as an alternative to PEG for microcell fusion. An expression vector encoding the MV-H and F genes was transfected into a CHO cell line which carries an artificial human chromosome (HAC) tagged with a drug-resistant selection marker. Functional expression of the transfected MV-H/F plasmids was confirmed by syncytium formation in a mixed culture of the engineered CHO cells with CD46-expressing HT1080 (fibrosarcoma) cells. Microcells were prepared from the donor cells and overlaid on HT1080, hiMSC (human immortalized mesenchymal stem cells), or HFL-1 (primary fibroblasts, derived from fetal lung) recipient cells. Drug-resistant colonies were obtained by selection culture and introduction of the HAC was confirmed by FISH analysis. The efficiency of microcell fusion with HT1080 or hiMSC using the MV fusogen was higher than that using PEG by an order of magnitude whereas the low efficiency of PEG-induced fusion with HFL1 was not improved by the MV fusogen. Difference in the fusion efficiency by the MV fusogen may be explained by the expression level of the CD46 receptor on these recipient cells. Potential extension of the host tropism of fusogenic microcells is also discussed.

## Results

### Introduction of the MV-F/H genes confers human cell-directed fusion ability on CHO cells

**Figure 2:**
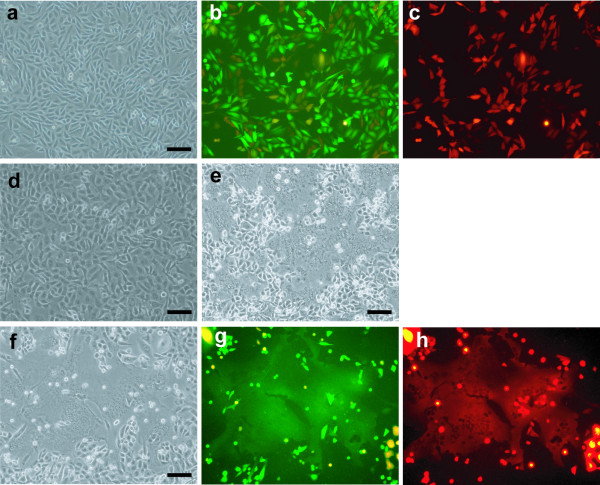
**Genetically engineered CHO cells can fuse with human cells**. G418-resistant CHO cells (a) expressed the GFP (b) and/or the fluorescent marker DsRed (c). An equal number of the CHO cells (a) and HT1080 cells (d) were mixed, and photographed 3 days later (f, g, h). Similar to the MV-F/H-transfected HT1080 cells (e), large multinucleated syncytia were observed (f). Syncytia made by heterofusion of CHO and HT1080 expressed GFP and DsRed fluorescence (g, h). The panels a, b, c and f, g, h show the same cell field, respectively. Scale bar, 10 μm. Photographs were taken under phase contrast (a,d,e,f) or fluorescence (b,c,g,h) microscopic conditions.

As the first step towards microcell fusion using an MV fusogen, we transfected expression cassettes encoding the MV-H and -F proteins, and the Neo/DsRed plasmid, into CHO(HAC) cells, which carry a HAC engineered from human chromosome 21 [14]. In the course of construction, the HAC was tagged with a Blasticidin-resistant (Bsr) gene and GFP. After transfection, the cells were cultured in medium containing the antibiotic G418. The G418-resistant cell population was recovered and designated as CHO4H6.1M. Most of them expressed both GFP and DsRed fluorescences (Figure 2a, b, c). It is known that transfection of MV-H/F into human cells induce syncytium formation by homofusion (Figure 2e). Unlike the case with MV-H/F transfected human cells, no syncytium was observed during propagation of the CHO4H6.1M cells (Figure 2a). The absence of syncytium formation was consistent with the prediction that the fusion machinery of the human specific-ecotropic MV does not function in rodent cells. The surface expression of the H protein was assessed by flowcytometry analysis. About one third of the cells showed slight expression of the H protein (additional file 1). It is generally expected that co-transfection of linearlized plasmids are concatenated and integrated together in the same chromosomal locus. Although the concomitant expression of H and F was not directly tested, a part of H protein expressing cells may also express F protein.

We then tested the functional expression of H and F on the cell surface. The CHO4H6.1M cells were co-cultured with the human tumor cell line HT1080. After culture for 24 h, a multinucleated syncytium was observed and further expanded (Figure 2f, g, h). Syncytia contained fluorescence-positive and -negative nuclei that were supposed to be derived from CHO and HT1080 cells, respectively (Figure 2h). The occurrence of heterofusion, and the absence of homofusion, suggested that although the transfected MV-H/F proteins did not induce fusion in the CHO cells, they were functionally expressed on the CHO cell surface and could mediate fusion with human cells.

### The genetically engineered CHO cells produce fusogenic microcells

**Figure 3:**
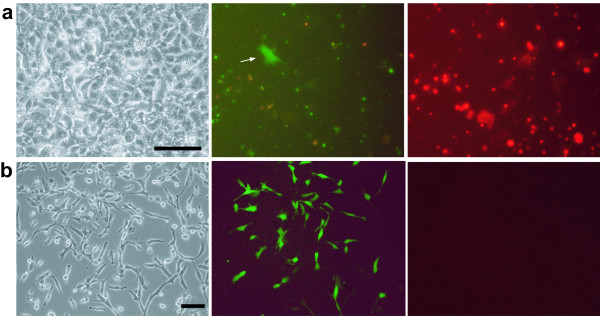
**Genetically engineered CHO cells can produce fusogenic microcells**. (a) Microcells were prepared from the MV-F/H-transfected CHO cells and placed on HT1080 cells. Photographs were taken after 24 h. A few recipient cells emitting fluorescence were observed. (b) HT1080 cells that had been in contact with the microcells were selected for 14 days with Blasticidin and drug-resistant colonies were photographed. These cells expressed GFP but not DsRed, suggesting retention of the HAC and elimination of MV-F/H-tagged host chromosomes. Photographs were taken under phase contrast (left) or fluorescence (center and right) microscopic conditions. Scale bar, 10 μm.

The ability of the CHO4H6.1M cells to function as donor cells for microcell fusion was then tested. Microcells were prepared from donor cells (~8 × 10^6^) that expressed DsRed and/or GFP (Figure 2a, b, c). These fluorescent microcells (~8 × 10^5^) were overlaid on 2 × 10^6 ^of HT1080 cells. Following 24 h incubation, weak fluorescence was detected in a few recipient cells (Figure 3a), while most of fluorescent microcells were retained as distinct particles. Fluorescence in recipient cells was explained by either transmission of the fluorescent protein from the microcells or de novo synthesis in microcell hybrids. To confirm that the donor chromosome was transferred, the cultures were expanded and treated with Blasticidin which selects for cells carrying tagged HAC. A total of 80 drug-resistant colonies were detected following Blasticidin selection. Selected colonies were picked up and propagated for the following analysis. Unlike the case with MV-H/F transfected human cells, no syncytium was observed during propagation of the Blasticidin-resistant clones.

**Figure 4:**
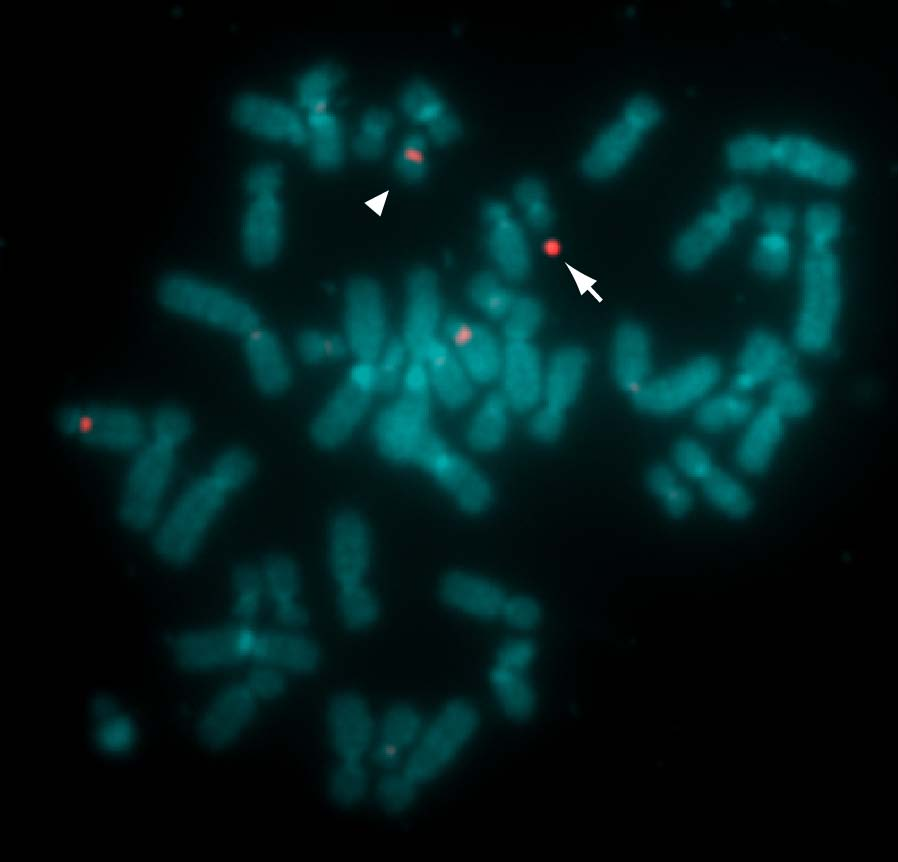
**Detection of chromosome transfer from the microcell to the donor cell**. Recipient cells exposed to the microcells, and selected by Blasticidin treatment, were analyzed by fluorescence in situ hybridization to detect cells containing the transferred HAC. The HAC is delineated using an alphoid satellite probe derived from human chromosome 21, and is shown in red (arrow). The alphoid satellite probe detects endogenous chromosomes 13 and 21 (arrowhead), in addition to the HAC, due to their sequence similarity.

FISH analysis was performed with the GFP expressing cells for the detection of the HAC transfer (Figure 4). Alphoid satellite probe derived from human chromosome 21 hybridized with endogenous chromsomes 21 and 13, due to their sequence similarity [14]. In addition to these endogenous chromosomes, the probe detected a minichromosome, which is not present in parental HT1080 cells (data not shown). Since the HAC was constructed by deleting almost all sequences from long and short arm of the chromosome 21, it retains alphoid satellite as substantial contents [14]. The estimated size of the HAC is less than 10 Mb, which corresponds to 1/5 of the original human chromosome 21. In comparison to the endogenous chromosome 21, the size of the minichromosome matched to the prediction. Obvious change in the karyotype of the host cells was not detected. These results demonstrated the introduction of the HAC by microcell fusion without disturbance of the host chromosomes.

It was noted that the colonies expressed GFP but not DsRed (Figure 3b). A possible explanation as to why the DsRed-expressing cells carrying HAC were not detected is that a microcell hybrid which received the donor chromosome tagged by DsRed/neo together with MV-H/F genes could not survive because of homofusion or fusion between surrounding cells. To test the elimination of the neo gene associated with the DsRed gene, Blasticidin-resistant clones were assessed for the sensitivity to G418 (additional file 2). All the clones tested were sensitive to G418. These results indicated that stable microcell hybrids were obtained by excluding the DsRed/neo-tagged donor chromosomes. These data proved that chromosome transfer was successfully achieved by microcell fusion via an MV fusogen.

### The MV fusogen is more efficient than PEG for microcell hybrid production

**Figure 5:**
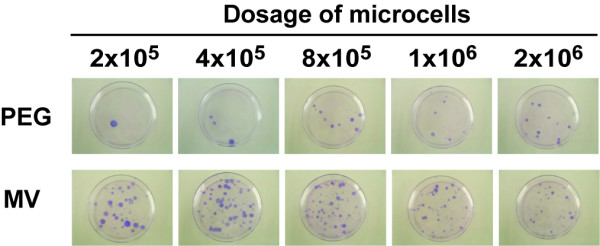
**The MV fusogen is more efficient than PEG for microcell hybrid production**. Microcells collected from 24 flasks (~5 × 10^6^) were fractionated into 5 dosing amounts (2 × 10^5^, 4 × 10^5^, 8 × 10^5^, 1 × 10^6^, 2 × 10^6^) and added to (MV), or fused with (PEG), 2 × 10^6 ^of HT1080 cells in a 60-mm dish. On the following day, fused cells were plated onto two 100-mm dishes. Following selection culture for 14 days, drug-resistant colonies were stained with Giemsa and photographed.

We next compared the efficiency of microcell fusion by MV fusogen with that by PEG. For this purpose, different amount of microcells were applied to the fixed number of recipient HT1080 cells. From 5 × 10^7 ^of CHO4H6.1M cells, ~5 × 10^6 ^of microcells were obtained. These microcells were aliquoted in five fractions with the ratio of 1,2,4,6,11, and applied to a fixed number (2 × 10^6^) of recipient cells. As a control, the conventional fusion experiment using PEG was performed with the parental HAC donor cell line CHOkkpqG4. Microcell fusion was determined as the number of drug-resistant colonies following selection culture with Blasticidin (Figure 5). The fusion efficiency by MV fusogen was consistently, and at most 10 times, higher than that by PEG (Table 1). Whereas fusion induced by PEG was gradually increased in a microcell dose-dependent manner, fusion induced by the MV fusogen did not show dose dependency. Input over threshold amount of microcells with MV fusogen seemed to reduce the appearance of microcell hybrids.

**Table 1:** Yield of drug-resistant microcell hybrids by using MV fusogen and PEG

Amount of applied microcells	Colony number^1^	
	
	MV	PEG
2 × 10^5^	51	5
4 × 10^5^	86	6
8 × 10^5^	94	15
1 × 10^6^	75	13
2 × 10^6^	60	22

1. Number of Blasticidin-resistant colonies obtained from 2 × 10^6 ^of HT1080 cells.

### Efficiency of microcell fusion via the MV fusogen differs with different recipient cells

**Figure 6:**
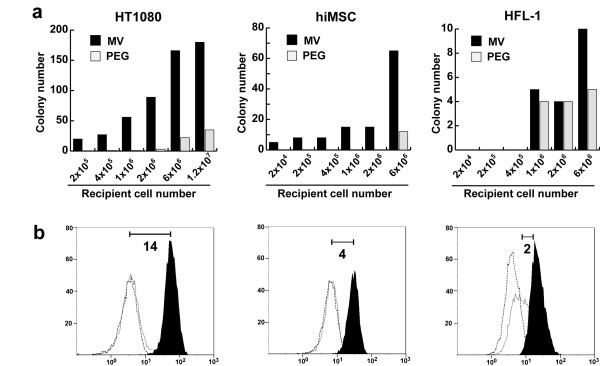
**The efficiency of microcell fusion correlated with the surface density of CD46 in recipient cells**. (a)A given number of microcells (~8 × 10^5^) were applied to different numbers of recipient cells and fused using MV or PEG. Abundant microcell hybrids, assessed as the number of drug-resistant colonies, were obtained using HT1080 and hiMSC cells. In contrast, few microcell hybrids were obtained using HFL-1 cells, irrespective of the fusogen used. (b) The expression level of CD46 on the cells was analyzed with flow cytometry by staining with FITC-conjugated anti-CD46 antibody (black peak) or an isotype control (white peak with solid line). No stained control was showed by white peak with dotted line. The numbers on the x and y axes represent fluorescence intensity and count of event, respectively. CD46 expression is indicated by the number within the graph which is the ratio of the mean fluorescence index of the black:white peaks.

We next determined whether the relatively high microcell fusion efficiency observed using the MV fusogen and HT1080 cells at optimal conditions could also be observed with other cell types. We therefore compared the fusion efficiency of the MV-fusion system using HT1080 with that using a mesenchymal stem cell line hiMSC and primary fibroblast cells HFL-1. A fixed number of microcells was applied to different numbers of recipient cells. After selection culture for 2 weeks, the number of drug-resistant colonies was counted (Figure 6a). Irrespective of the number of recipient cells, MV-fusion consistently induced more resistant colonies than PEG fusion in the HT1080 cell cultures. Although the colony number obtained by MV-fusion in hiMSC cells was approximately half of that in HT1080 cells, the hiMSC were also responsive to MV fusion. In contrast, difference in an order of magnitude was not observed between MV- and PEG-induced resistant colonies using the HFL-1 cells. The PEG-induced fusion efficiency using HFL-1 cells was quite low compared to the other cell lines and this low level of fusion was not overcome using the MV fusogen.

In HT1080 and hiMSC, it was noted that MV fusion induced resistant colonies in small numbers of recipient cells in which no colonies were induced by PEG fusion. Fusion efficiency defined as frequency of drug resistant-colony formation per recipient cells scored highest (~10^-4^), when small number of recipient cells (2 × 10^4^) were used. In HT1080 and hiMSC, the maximum efficiency was 50 and 100 times greater than that using conventional PEG fusion, respectively.

**Figure 7:**
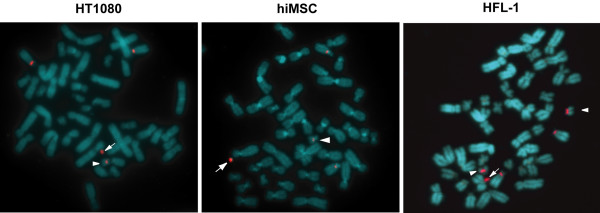
**Detection of transferred HAC in microcell hybrids by FISH**. The transferred HAC (arrow) is delineated by fluorescence in situ hybridization using an alphoid satellite probe derived from human chromosome 21. The alphoid satellite probe (red) detects endogenous chromosomes 13 and 21(arrowheads), in addition to the HAC, due to their sequence similarity. Results from HT1080, hiMSC, or HFL-1 cells were placed on left, center, and right, respectively.

For drug-resistant cells, FISH analysis was performed for the detection of transferred HACs (Figure 7). In addition to the endogenous chromosomes 21 and 13, the alphoid satellite probe detected a minichromosome, which is not present in parental HT1080, hiMSC, and HFL-1 cells. In comparison to the endogenous chromosome 21, the size of the minichromosomes matched to the prediction. In HFL-1, the alphoid probe detected a pair of endogenous chromosomes 21 and 13, whereas in HT1080 and hiMSC, the probe detected a single chromosome 21. While HFL-1 is primary cells, HT1080 and hiMSC were immortalized by the transformation [27]. Aberrant chromosome number in HT1080 and hiMSC may be explained by the transformation. The result obtained by the PEG fusion (data not shown) was consistent with that of the MV fusion. These results demonstrated that the HAC was transferred to the recipient cells by microcell fusion without disturbance of the host chromosomes.

### Efficiency of microcell fusion may depend on the surface density of the CD46 receptor

MV is known to be a potent and specific oncolytic agent that causes cytopathic effects due to extensive syncytium formation. A previous study reported that the extent of intercellular fusion in MV-infected human cells including HT1080, was determined by the surface density of CD46 [28]. These data suggested that the extent of microcell fusion may also be affected by the surface density of CD46. Therefore, we measured the surface density of CD46 in the three cell lines using flow cytometry. The surface density of CD46 was quantified and expressed as the ratio of the mean fluorescence index of anti-CD46 antibody-stained and isotype control-stained cells. The HT1080, hiMSC, and HFL-1 cells showed high, moderate, and low levels of CD46 surface expression, respectively (Figure 6b). A correlation was noted between fusion efficiency and expression level of CD46. These results is consistent with the previous report of intercellular fusion in MV-infected human cells that cell fusion was minimal at low receptor densities but increased significantly above a threshold density of the receptor [28]. Therefore, measurement of the surface density of CD46 is a useful criterion for prediction of the ability of the MV fusogen to induce microcell fusion in recipient cells.

## Discussion

We aimed to determine whether microcell fusion could be achieved using an MV fusogen. We showed that introduction of genes encoding MV envelope proteins enables rodent cells to produce fusogenic microcells that efficiently transmit donor chromosomes to recipient human cells expressing a high level of CD46 (Figure 1). This method has several advantages over the conventional PEG-fusion method for chromosome transfer. First, microcell hybrids can be obtained from a low number of recipients, even from recipients that are too low in number for hybrids to be obtained by PEG-fusion, as long as the recipients express the CD46 receptor over a threshold density. Second, the procedure for microcell fusion is extremely simple. Thus the formation of microcell hybrids requires only the addition of prepared microcells to recipient cells. This ease of application, which avoids the laborious tasks of handling a highly viscous PEG solution and performing repeated washout steps, may improve the reproducibility of microcell fusion. We obtained abundant microcell hybrids using MV-fusion even using donor cells that should have heterogeneity in copy number of the harbored MV-F/H expression plasmids. Further improvement in the fusion efficiency can be expected by clonal selection of donor cells with a high level of MV-F/H expression.

Application of microcells over a threshold amount inversely reduced the appearance of drug-resistant colonies. In principle, the collected microcells are composed of a heterogeneous population and the nature of the contained chromosome is not uniform. Hence, a microcell containing HAC is expected to yield drug-resistant colonies. In contrast, a microcell containing the MV-F/H-tagged donor chromosome can potentially induce secondary fusion between the microcell hybrid and the surrounding recipient cells, which ultimately reduces the survival of microcell hybrids carrying the HAC. Application of lower numbers of microcells may help to obviate the negative effects that occur due to carry-over of the genes encoding the MV-H/F proteins.

The next issue for microcell fusion via an MV fusogen is whether the tropism for recipient cells can be altered from the default CD46 receptor to arbitrary receptors. Engineering of viral tropism has been pursued for many gene therapy-based strategies [29-31]. An MV-Edm-reverse genetics system has allowed the design and construction of recombinant MVs that are better suited for oncolytic applications. Retargeting of MV has been achieved by the addition of specificity domains to the extracellular (carboxyl) H-protein terminus. Thus, the addition of EGF or IGF1 to the H protein efficiently retargeted MV to CD46-negative rodent cells expressing the human EGF or IGF1 receptor, and caused extensive syncytium formation [32]. Furthermore, the addition of single chain antibody fragments to the H protein also efficiently retargeted MV to the EGF receptor, myeloma surface antigen CD38, or urokinase receptor [25,26,33]. These results suggest that chimeric H proteins containing an additional protein domain are functionally displayed on the surface of transduced cells. Thus these chimeric H proteins sustain the ability to interact with the targeted receptor, and can proceed to membrane fusion by cooperating with the F protein. Therefore, modification of the H protein potentially enables retargeting of fusogenic microcells to recipient cells of interest.

## Conclusions

The MV-fusogen procedure for microcell fusion that has been demonstrated in this study, not only facilitates microcell fusion but also facilitates future manipulation of the cell targeting of microcells, by exploitation of previously established genetic engineering of the MV-H protein.

## Methods

### Cell culture

HT1080, and hiMSC cells were grown in Dulbecco's modified Eagle's medium (Sigma) supplemented with 10% fetal bovine serum (BioWest). HFL-1, obtained from the Riken cell bank, was grown in Ham's F12 medium (Sigma) supplemented with 15% fetal bovine serum. HAC donor CHO cells were grown in Ham's F-12 medium supplemented with 10% fetal bovine serum.

### MV envelope protein expression plasmids and transfection

Construction of the measles H protein expression plasmid pTNH6-H was previously described [20]. The plasmid pCAG-T7-F encodes the F protein of the Edmonston-B strain under the control of CAG promoter (Nakamura, unpublished). The DsRed/neo expression plasmid, pDsRed-Monomer-N1, was purchased from Clontech. Plasmids were linearized by restriction digestion with PvuI (NEB) before transfection. HAC donor CHO cells (8 × 10^4^/well in 24-well plate (Nunc))were co-transfected with 0.3 μg each of pTNH6-H and pCAG-T7-F, and 0.25 μg of pDsRed-Monomer-N1 using cationic lipid (Lipofectamine 2000; Invitrogen). At 24 h after transfection, the cells were re-plated at low density and selected for 14 days with 800 μg/ml of G418 (Nacalai). Drug-resistant cells were recovered as a mixed population.

HT1080 cells (2 × 10^6^/6 cm dish) were co-transfected with 0.4 μg each of pTNH6-H and pCAG-T7-F using cationic lipid (Lipofectamine 2000). After culture for 6 h, syncytium formation was tested under microscope.

### Co-culture assay

Equal numbers (1 × 10^5^) of G418-resistant CHO cells and HT1080 cells were plated in a 60-mm dish (Beckton Dickinson) and cultured for a period of 3 days and syncytium formation was determined microscopically.

### Microcell fusion

The day before microcell fusion, recipient cells were trypsinized and counted. A given number of recipient cells (2 × 10^4^, 2 × 10^5^, 4 × 10^5^, 1 × 10^6^, 2 × 10^6^, or 6 × 10^6^) were plated in adequate culture vessel (96-well, 24-well, 12-well, 6-well, 60-mm diameter, and 100-mm diameter, respectively). Donor CHO cells, grown in T-25 flasks (Nunc), were treated with 0.1 μg/ml colcemid (Gibco) for 72 h to induce micronuclei formation. Flasks were filled with medium containing 10 μg/ml cytochalasin B (Sigma) and centrifuged for 1 h at 8,000 rpm (11,899 ×g) in a JLA-10.5 rotor (Beckman). Pellets containing a crude microcell preparation were recovered and passed through membranes of 8-, 5-, and 3-μm pore size (Whatman). Collected microcell preparations were used for fusion with recipient cells. For PEG fusion, microcells were suspended in DMEM medium (Sigma) containing 50 μg/ml PHA-P (Difco) and added to the recipient cells. After incubation at 37°C for 15 min, the medium was discarded. The cells were then exposed to 50% (w/v) PEG1500 (Roche) for 1 min and washed three times with serum free medium. On the day following PEG treatment, the cells were trypsinized, sparsely replated, and cultured for 14 days in medium with 3 μg/ml of Blasticidin (Funakoshi). For MV fusion, an aliquot of microcells prepared from CHO4H6.1M was overlaid on recipient cells and left for 24 h. The cells were then trypsinized, sparsely replated, and cultured for 14 days in medium with 3 μg/ml of Blasticidin.

### FISH analysis

Metaphase chromosomes were prepared from colcemid-treated cell cultures by hypotonic treatment with 0.075 M KCl and methanol/acetate (3:1) fixation. Fluorescence in situ hybridization was carried out using the alphoid DNA probe p11-4 labeled with digoxigenin (Roche) [34]. The digoxigenin signal was detected with an anti-digoxigenin-rhodamine complex (Roche). The chromosomes were counterstained with DAPI (Sigma). Photographs were taken using a CCD camera mounted on a fluorescence microscope (Nikon). Images were processed using the software attached to the microscope.

### Flow cytometry analysis

Cells were dispersed by treatment with 0.2% EDTA/PBS, washed twice with PBS, and resuspended in ice-cold PBS containing 2% (w/v) BSA at a concentration of 10^6 ^cells/ml. The cells were then incubated for 60 min on ice with a 1:50 final dilution of FITC-labeled anti-CD46 antibody (clone E4.3; BD Pharmingen), or FITC-labeled isotype control (clone G155-178; BD Pharmingen). After washing with BSA/PBS, the cells were analyzed by flow cytometry using an EPICS ALTRA (Beckman Coulter).

## Authors' contributions

MK designed the study, performed plasmid transfections and cell fusion assays, and drafted the manuscript. YK designed the study and helped to draft the manuscript. KK, NK and MT performed microcell fusions and FISH analyses. YN performed flow cytometry analyses. TN provided materials and contributed to the conception of the study. MO conceived of and supervised the study. All authors read and approved the final manuscript.

## Supplementary Material

Additional file 1 **The Measles H protein is expressed on the surface of the genetically engineered CHO cells**. The surface expression of measles H protein on the CHO4H6.1M cells was analyzed by flowcytometry. The cells were stained with anti-measles H and AlexaFluor 647 secondary antibody (grey peak) or only secondary antibody (white peak with solid line). No stained control was showed by white peak with dotted line. Methods. Cells were dispersed by treatment with 0.2% EDTA/PBS, washed twice with PBS, and resuspended in ice-cold PBS containing 2% (w/v) BSA at a concentration of 10^6 ^cells/ml. The cells were then incubated for 60 min on ice with a 1:150 final dilution of the primary mouse monoclonal ascites antibody recognizing measles H protein (Clone CV1, CV4; Chemicon). Subsequently, the cells were washed with 2% (v/v) FBS/PBS and incubated for for an additional 30 min with 1:250 final dilution of Alexa Flour 647 conjugated goat anti-mouse IgG (Molecular Probes). After washing with BSA/PBS, the cells were analyzed by flow cytometry using MoFlo XDP (Beckman Coulter). Click here for file

Additional file 2 **Microcell hybrids excluded the neo gene-tagged donor chromosome**. Microcell hybrids obtained by Blasticidin selection were assessed for sensitivity to G418. 10^4 ^hybrid cells were plated in 12 well cluster and cultured for one week with or without antibiotics. Cells were fixed by methanol, stained with Giemza, and photographed. Microcell hybrids were sensitve to G418, indicating that the neo gene-tagged donor chromosome was eliminated from microcell hybrids. Click here for file
