# Supplementary figures and images for: Exploitation of the interaction of measles virus fusogenic envelope proteins with the surface receptor CD46 on human cells for microcell-mediated chromosome transfer
Source: BMC Biotechnol. 2010 May 6;10:37. doi: 10.1186/1472-6750-10-37 (PMC2874513; doi:10.1186/1472-6750-10-37)

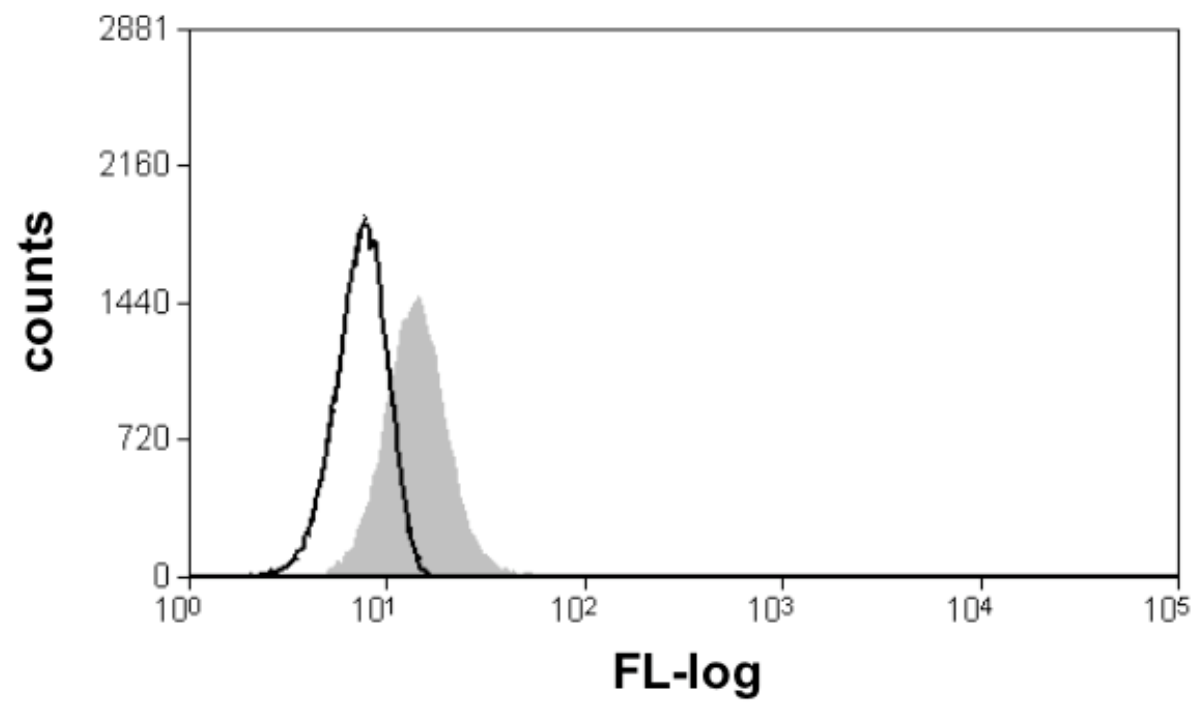

Supplement: Additional file 1 — The Measles H protein is expressed on the surface of the genetically engineered CHO cells. The surface expression of measles H protein on the CHO4H6.1M cells was analyzed by flowcytometry. The cells were stained with anti-measles H and AlexaFluor 647 secondary antibody (grey peak) or only secondary antibody (white peak with solid line). No stained control was showed by white peak with dotted line. Methods. Cells were dispersed by treatment with 0.2% EDTA/PBS, washed twice with PBS, and resuspended in ice-cold PBS containing 2% (w/v) BSA at a concentration of 106 cells/ml. The cells were then incubated for 60 min on ice with a 1:150 final dilution of the primary mouse monoclonal ascites antibody recognizing measles H protein (Clone CV1, CV4; Chemicon). Subsequently, the cells were washed with 2% (v/v) FBS/PBS and incubated for for an additional 30 min with 1:250 final dilution of Alexa Flour 647 conjugated goat anti-mouse IgG (Molecular Probes). After washing with BSA/PBS, the cells were analyzed by flow cytometry using MoFlo XDP (Beckman Coulter). [file 1472-6750-10-37-S1.PDF]

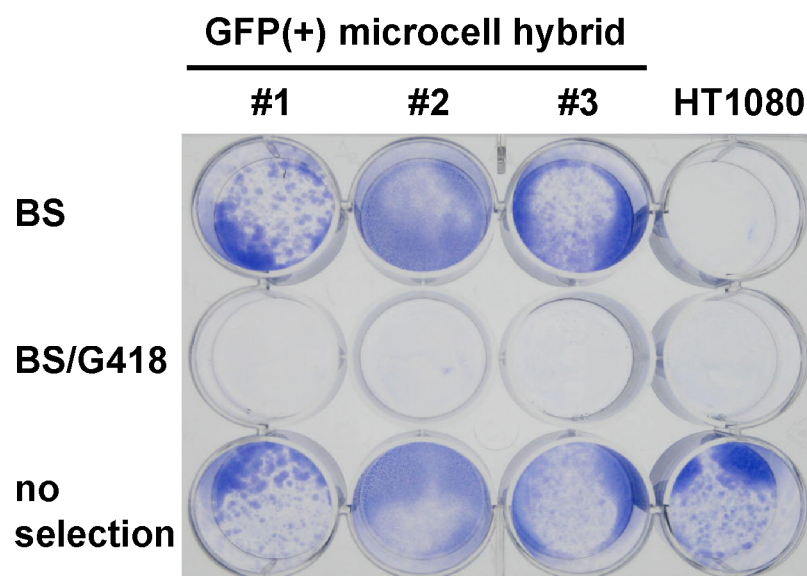

Supplement: Additional file 2 — Microcell hybrids excluded the neo gene-tagged donor chromosome. Microcell hybrids obtained by Blasticidin selection were assessed for sensitivity to G418. 104 hybrid cells were plated in 12 well cluster and cultured for one week with or without antibiotics. Cells were fixed by methanol, stained with Giemza, and photographed. Microcell hybrids were sensitve to G418, indicating that the neo gene-tagged donor chromosome was eliminated from microcell hybrids. [file 1472-6750-10-37-S2.PDF]
